# Quantitative photoacoustic endoscopy reveals early microvascular biomarkers of endometrial fibrosis in a rat model

**DOI:** 10.1016/j.isci.2026.117609

**Published:** 2026-09-28

**Authors:** Xiaowen Liang, Zhili Guo, Kedi Xiong, Zijian He, Dongyu Zhang, Shihan Kang, HongJi Chen, Meng Du, Xiaoyan Kui, Xue Wen, Zhiyi Chen

**Affiliations:** 1 School of Computer Science and Engineering, Central South University, Changsha 410083, China; 2 Key Laboratory of Medical Imaging Precision Theranostics and Radiation Protection, College of Hunan Province, the Affiliated Changsha Central Hospital, Hengyang Medical School, University of South China, Changsha 410004, China; 3 Institute of Medical Imaging, Hengyang Medical School, University of South China, Hengyang 421001, China; 4 Institute for Future Sciences, University of South China, Changsha 410008, China; 5 College of Biophotonics, School of Optoelectronic Science and Engineering, South China Normal University, Guangzhou 510631, China; 6 Department of Ultrasound Medicine, Department of Medical Imaging, The Affiliated Changsha Central Hospital, Hengyang Medical School, University of South China, Changsha 410004, China; 7 State Key Laboratory of Precision Electronics Manufacturing Technology and Equipment, Guangdong University of Technology, Guangzhou 510006, China

**Keywords:** photoacoustic endoscopy, endometrial fibrosis, endometrial injury, microvascular dynamics, imaging biomarker

## Abstract

Endometrial fibrosis, the core pathological feature of intrauterine adhesion, lacks effective early assessment methods. This study used a quantitative photoacoustic endoscopy (PAE) method to assess the relationship between early PAE-derived features and late-stage fibrosis by characterizing microvascular and inflammatory dynamics in the injured endometrium. PAE captured dynamic endometrial microvascular perfusion changes post-injury, with distinct late vascular remodeling patterns under mild and severe injury. PAE-derived vessel area and mean E lacunarity correlated strongly with histologically validated vascular density (r = 0.90 and −0.84, respectively), and at mid-to-later stages, PAE features correlated with inflammatory cell counts. Day 7 post-injury emerged as a key evaluation window, with vessel area showing AUCs of 0.938 for distinguishing mild fibrosis and 0.946 for severe fibrosis. These findings suggest that quantitative PAE provides high-resolution, biopsy-free monitoring of the endometrial microenvironment and may offer microvascular biomarkers that link early imaging changes to subsequent fibrosis progression.

## Highlights

• Quantitative PAE enables longitudinal monitoring of endometrial microvascular changes
• PAE-derived vessel area and lacunarity correlate with histological vascular density
• PAE-derived microvascular features are associated with inflammatory infiltration
• Day 7 PAE defines the optimal window for evaluating endometrial fibrosis

## Graphical abstract

**Figure:**
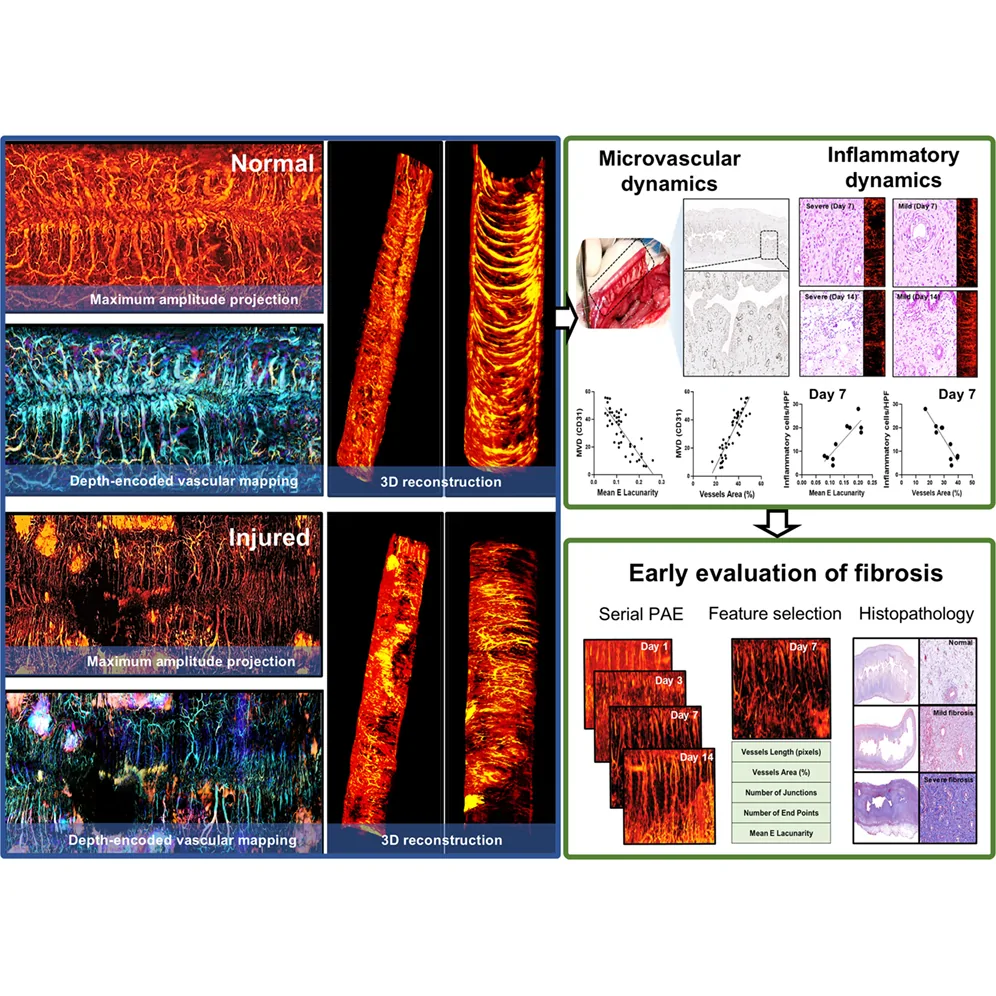

## Teaser

Endocrinology; Clinical endocrinology; Laboratory endocrinology; Medical endocrinology

## Introduction

Endometrial fibrosis, the core pathological feature of intrauterine adhesion (IUA), typically develops following endometrial injury and leads to loss of endometrial function and infertility.^1^ With the increasing incidence of intrauterine procedures and associated endometrial injuries, the occurrence of endometrial fibrosis has increased. Epidemiological studies suggest that up to half of women who undergo intrauterine procedures may develop varying degrees of endometrial fibrosis following intrauterine interventions such as induced abortion or hysteroscopic surgery.^2^^,^^3^ Without timely and effective intervention, this pathological fibrotic change may persist and ultimately lead to loss of reproductive function.^4^ In addition, unnecessary or excessive interventions may adversely affect reproductive potential, especially for patients who have the prospect of achieving functional repair of the endometrium.^5^ Therefore, early and accurate evaluation of postinjury fibrosis is of clinical importance, as it can guide timely intervention to delay fibrosis progression in high-risk individuals, thereby reducing the risk of fertility loss while sparing low-risk individuals from unnecessary interventions.

Spatiotemporal monitoring of the postinjury microenvironment—particularly microvascular and inflammatory dynamics—is essential for understanding and evaluating fibrosis progression. The pathogenesis of fibrosis in the postinjury endometrial microenvironment is a dynamic spatiotemporal process involving chronic inflammation, microvascular dysfunction, and abnormal extracellular matrix deposition.^6^^,^^7^ Following injury, inflammatory cells are recruited to the local injury region and sustain a profibrotic milieu through the continuous release of cytokines and growth factors. Concurrently, damage to the microvasculature induces local ischemia and hypoxia, which further exacerbates fibroblast activation and matrix deposition.^8^ These intertwined processes critically determine the ultimate fibrotic outcome. However, current clinical tools for this purpose are limited. While hysteroscopy, as the first-line diagnostic approach, provides direct visualization of structural abnormalities, it does not adequately capture subtle functional changes in the microenvironment and is associated with risks of further iatrogenic injury or recurrence.^9^^,^^10^ Transvaginal ultrasound serves as a noninvasive alternative for assessing global structure and blood flow, but its utility is constrained by limited imaging resolution.^11^ Although advanced techniques such as contrast-enhanced ultrasound^12^ and super-resolution ultrasound^13^ offer improved image resolution, their indirect, extracavitary approach limits spatial resolution for detailed endometrial assessment within the uterine cavity. Moreover, these high-resolution imaging techniques rely on the extraneous injection of contrast agents, and their safety for reproductive function requires further investigation. These limitations highlight the need to develop an intrauterine minimally invasive monitoring method with high spatiotemporal resolution for the early evaluation of endometrial fibrosis following injury.

Photoacoustic imaging (PAI) combines the high contrast of optical imaging with the deep penetration of ultrasound, providing a high-resolution modality for microcirculation assessment and visualization.^14^ Building on high-resolution multifunctional microvascular imaging, PAI has shown promise in detecting inflammatory activity,^15^ localizing tissue damage,^16^ and classifying fibrosis severity.^17^ As an extension into luminal organs, photoacoustic endoscopy (PAE) integrates miniaturized optical and acoustic components into an endoscopic platform, enabling label-free, high-resolution intraluminal imaging with minimal procedural damage.^18^^,^^19^^,^^20^ Previous work from our group established the ability of intrauterine PAE to image the microvascular network of the normal rat endometrium.^21^ Nevertheless, how early postinjury microvascular dynamics evolve and contribute to the pathogenesis of endometrial fibrosis, and whether PAE can help elucidate these mechanisms, have yet to be explored. In this study, we established a PAE-based methodology for the spatiotemporal monitoring of the postinjury endometrial microenvironment for early evaluation of fibrosis (Figure 1). By performing serial PAE imaging, we tracked the dynamic changes in the endometrial microvasculature and inflammatory burden following injury, thereby establishing temporal and spatial correlations between PAE-derived features and histopathological outcomes. On the basis of the characteristic “microperfusion-inflammation-fibrosis” progression within the postinjury endometrial microenvironment, we analyzed the correlation between early-to-mid-phase PAE features and late-stage fibrosis outcomes. This approach may improve our understanding of the pathophysiology of endometrial fibrosis in preclinical models, potentially informing future clinical strategies.

**Figure 1:**
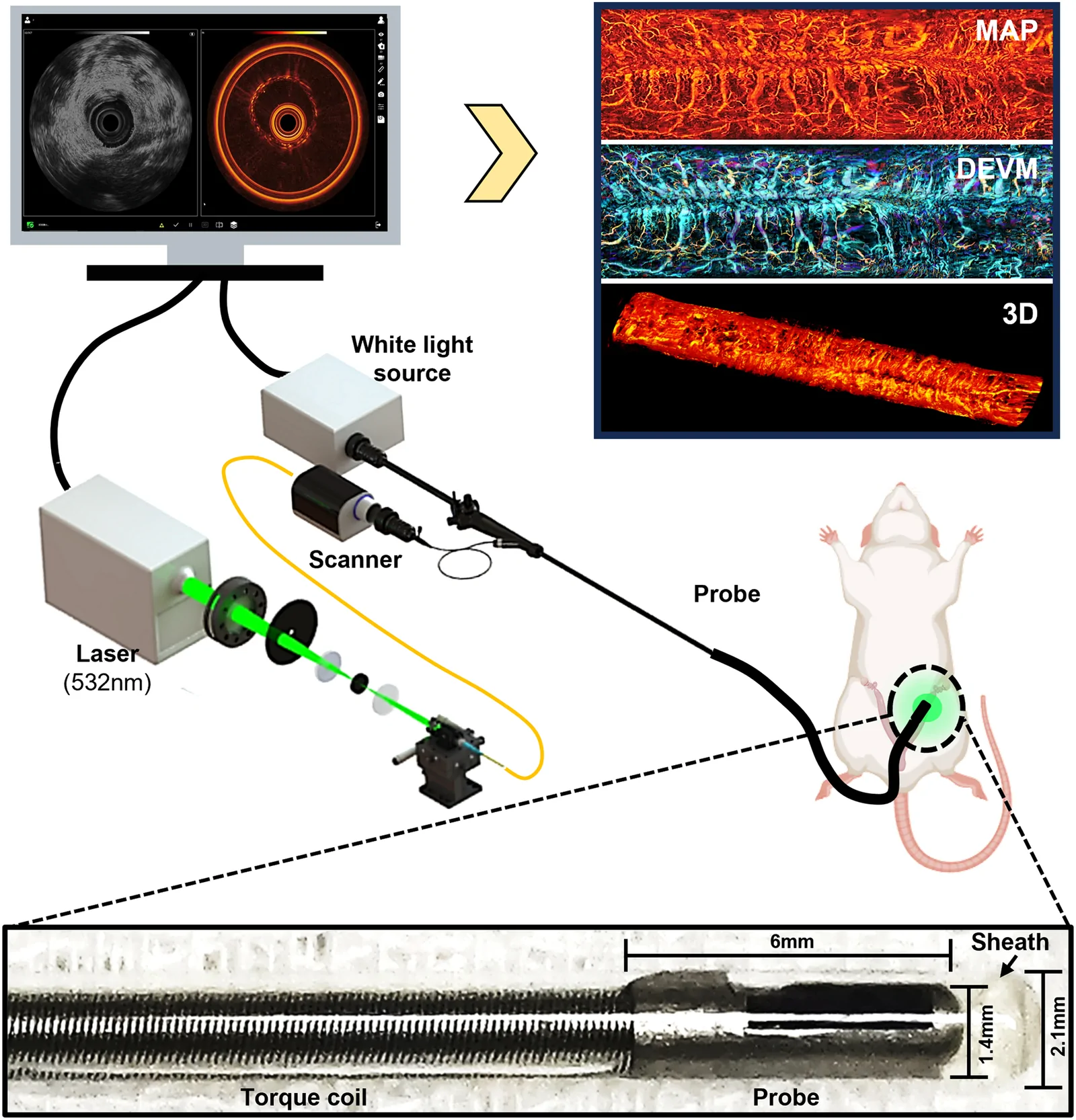
Experimental schematic of *in vivo* intrauterine PAE

## Results

### Assessing injury severity through serial PAE evaluation of endometrial microvascular dynamics

Quantitative intrauterine PAE was used to assess the dynamic changes in the endometrial microvasculature of mild/severely injured IUA model rats. Maximum amplitude projection (MAP) images revealed a temporary increase in distal branch microvessels and increased PA signals on day 1 in both groups, followed by a decrease in photoacoustic signals and progressive loss of microvascular regularity and continuity from day 3 to day 14 in severe injury rats (Figure 2A). In the mild injury group, the initial damage was followed by microvascular functional reconstruction between 7 and 14 days (Figure 2B). Depth-encoded vascular mapping (DEVM) images revealed increased numbers of distal branch microvessels on day 1, which is consistent with inflammatory stimulation. These appeared on the DEVM images as tiny, disordered vessels located closer to the probe.

**Figure 2:**
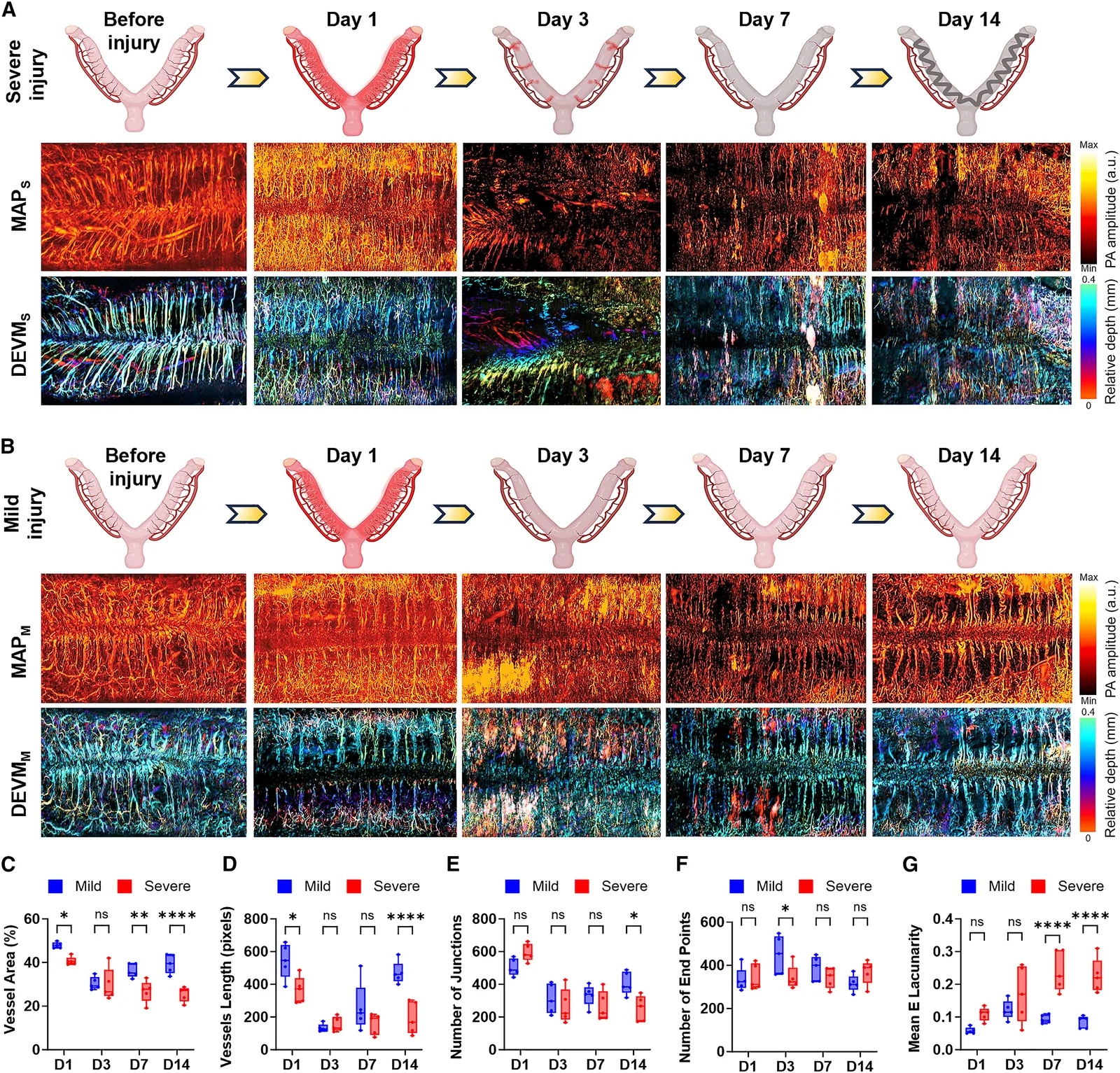
PAE-based serial tracking of microvascular dynamics following mild and severe endometrial injury (A and B) Representative longitudinal PAE-derived MAP and DEVM images from rats with severe (A) or mild (B) endometrial injury. (C–G) Quantitative comparison of microvascular parameters between the mild and severe injury groups across the study time course, including (C) vessel area, (D) vessel length, (E) number of junctions, (F) number of endpoints, and (G) mean E lacunarity. The data are presented as mean ± SD, *n =* 5 biological replicates. ∗*p* < 0.05, ∗∗*p* < 0.01, ∗∗∗∗*p* < 0.0001 by two-way ANOVA followed by Tukey’s post hoc test; ns, not significant.

Vascular segmentation was visualized and quantitative metrics were measured using AngioTool (Table 1). Compared with those in normal rats, the vessel area and vessel length in mild injury rats changed little on day 1 but then decreased during the early stage (days 1–3), indicating damage to the vascular network. During the same period, the decrease in the total number of junctions and the increase in the total number of endpoints suggested impaired vascular integrity and possible disruption of blood flow. In the mild injury group, the vessel area, vessel length, number of endpoints, and lacunarity gradually returned to near-normal levels by day 14, suggesting functional vascular recovery. In contrast, severe injury resulted in more extensive microvascular damage. This was reflected in a rapid decrease in vessel area and length during the early stage (days 1–7), followed by a sustained increase in lacunarity during the later stage. Elevated lacunarity suggested increased morphological complexity and further vascular deterioration. Given the changes in the PAE images, we further investigated the ability of effective PAE imaging features to distinguish the severity of injury. The effective mild/severe injury assessment parameters at the early stage (days 1–3) including the vessel area, vessel length, and number of endpoints, are shown in Figures 2C–2G. The effective parameters at the later stage of injury (days 7–14) included the vessel area, vessel length, number of junctions, and mean E lacunarity.

**Table 1:** PAE imaging features analyzed in different groups

Variables	Normal	Injury severity	Day 1	Day 3	Day 7	Day 14
Vessel length (pixels)	570.69 ± 231.67	Mild	503.88 ± 213.12	133.19 ± 31.01^a^^,^^b^	258.32 ± 146.35^c^	470.11 ± 169.96^c^^,^^d^
		Severe	367.23 ± 133.83	153.81 ± 46.87^a^^,^^b^	158.55 ± 86.24^a^^,^^b^	193.12 ± 99.31^a^^,^^b^
Vessel area (%)	42.13 ± 3.49	Mild	47.90 ± 3.48^a^	30.44 ± 3.06^a^^,^^b^	36.30 ± 3.98^b^^,^^c^	39.37 ± 5.12^b^^,^^c^
		Severe	40.69 ± 6.99	29.84 ± 6.91^a^	30.17 ± 7.44^a^^,^^b^	25.57 ± 4.20^a^^,^^b^
Number of junctions	510.30 ± 111.68	Mild	504.30 ± 101.92	311.20 ± 93.32^a^^,^^b^	332.50 ± 80.46^a^^,^^b^	407.50 ± 94.20^a^^,^^b^^,^^d^
		Severe	594.60 ± 129.03	265.60 ± 119.02^a^^,^^b^	265.20 ± 110.02^a^^,^^b^	253.60 ± 87.52^a^^,^^b^
Number of endpoints	301.80 ± 63.20	Mild	333.70 ± 61.32	468.60 ± 134.40^a^^,^^b^	385.40 ± 77.75	316.40 ± 48.58
		Severe	344.60 ± 76.95	342.10 ± 71.19	343.40 ± 68.76	369.00 ± 72.21
Mean E lacunarity	0.08 ± 0.03	Mild	0.06 ± 0.02	0.12 ± 0.03^b^	0.09 ± 0.01^b^	0.09 ± 0.03^b^
		Severe	0.12 ± 0.04	0.17 ± 0.09	0.24 ± 0.07^a^^,^^b^	0.23 ± 0.06^a^^,^^b^

Data are presented as the means ± SDs. Statistical differences between groups were assessed with one-way ANOVA, *n* = 5 biological replicates.

a *p* < 0.05 versus the control group.

b *p* < 0.05 versus the 1-Day group.

c *p* < 0.05 versus the 3-Day group.

d *p* < 0.05 versus the 7-Day group.

### Monitoring of endometrial microvascular dynamics and characterization of angiogenesis by PAE with histopathological correlation

In this study, PAE enabled the visualization of key histopathological features, including vascular injury, angiogenesis, and mural blood clots, within the postinjury endometrial microenvironment. 3D-PAE and MAP images revealed the spatial orientation of pathological changes. In parallel, the DEVM images visualize the depth and extent of angiogenic regions (Figures 3 and S1).

**Figure 3:**
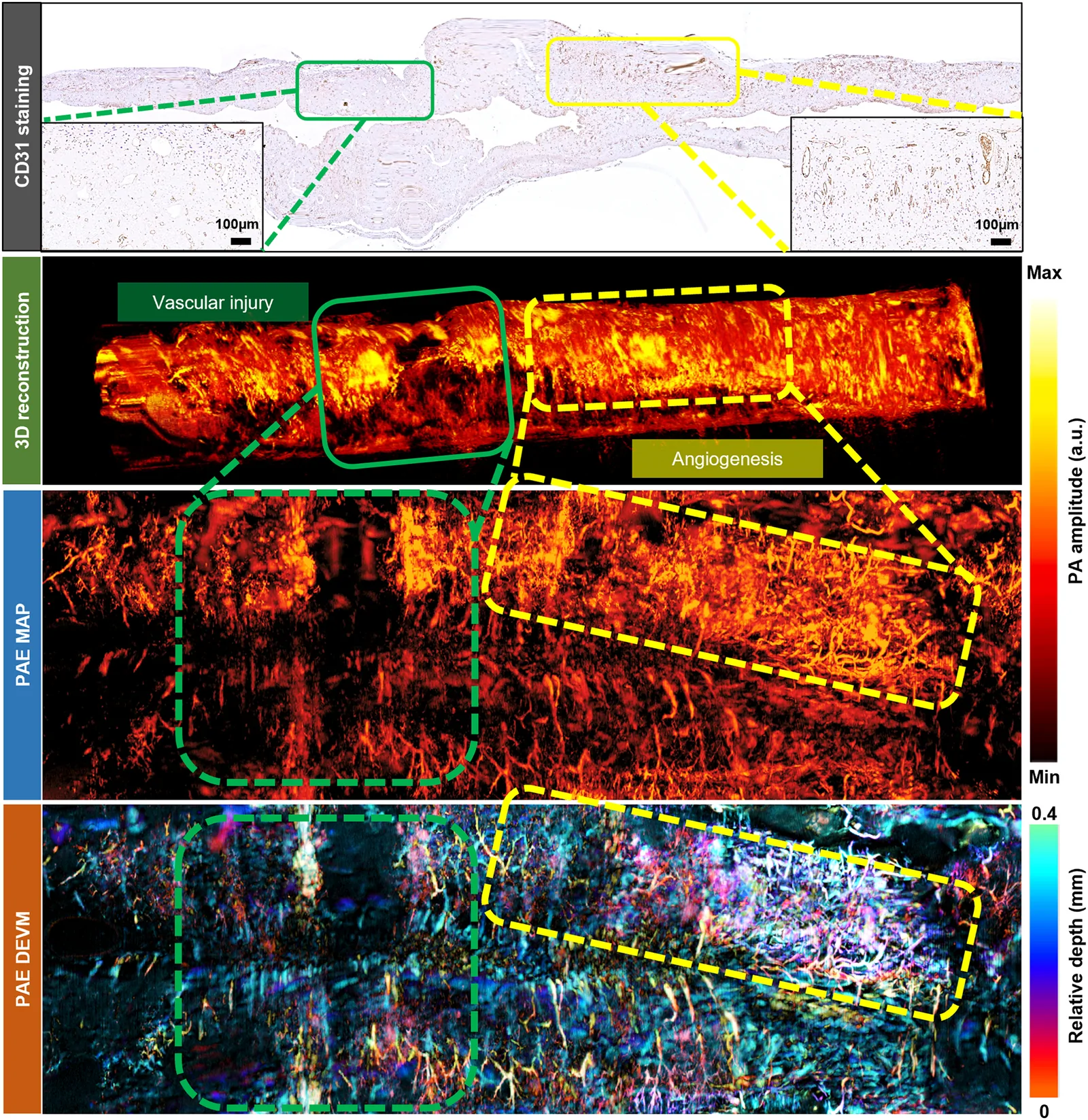
2D and 3D PAE imaging of endometrial microvascular injury and angiogenesis on day 14 PAE visualizes areas of vascular injury (green box) and angiogenesis (yellow box), which correspond spatially to regions highlighted by CD31 immunohistochemistry. Scale bars, 100 μm.

To validate the ability of PAE to characterize microvascular dynamics at the histological level, we correlated quantitative PAE imaging features (Figure 4A) with the expression of CD31—a specific vascular endothelial marker—across postinjury stages. CD31 immunohistochemistry was quantified as the microvessel density (MVD) in a high-power field (400×). In the mild injury group, the MVD showed a transient increase on day 1, followed by vascular damage on day 3, before stabilizing and trending toward normalization from day 7–14, accompanied by structural recovery (e.g., gland and epithelium regeneration). Conversely, in the severe injury group, CD31 staining revealed a time-dependent decrease in endometrial MVD, indicating progressive impairment of vascular regeneration (Table 2). Representative CD31 staining results are shown in Figures 4B and 4C for the mild and severe groups, respectively.

**Figure 4:**
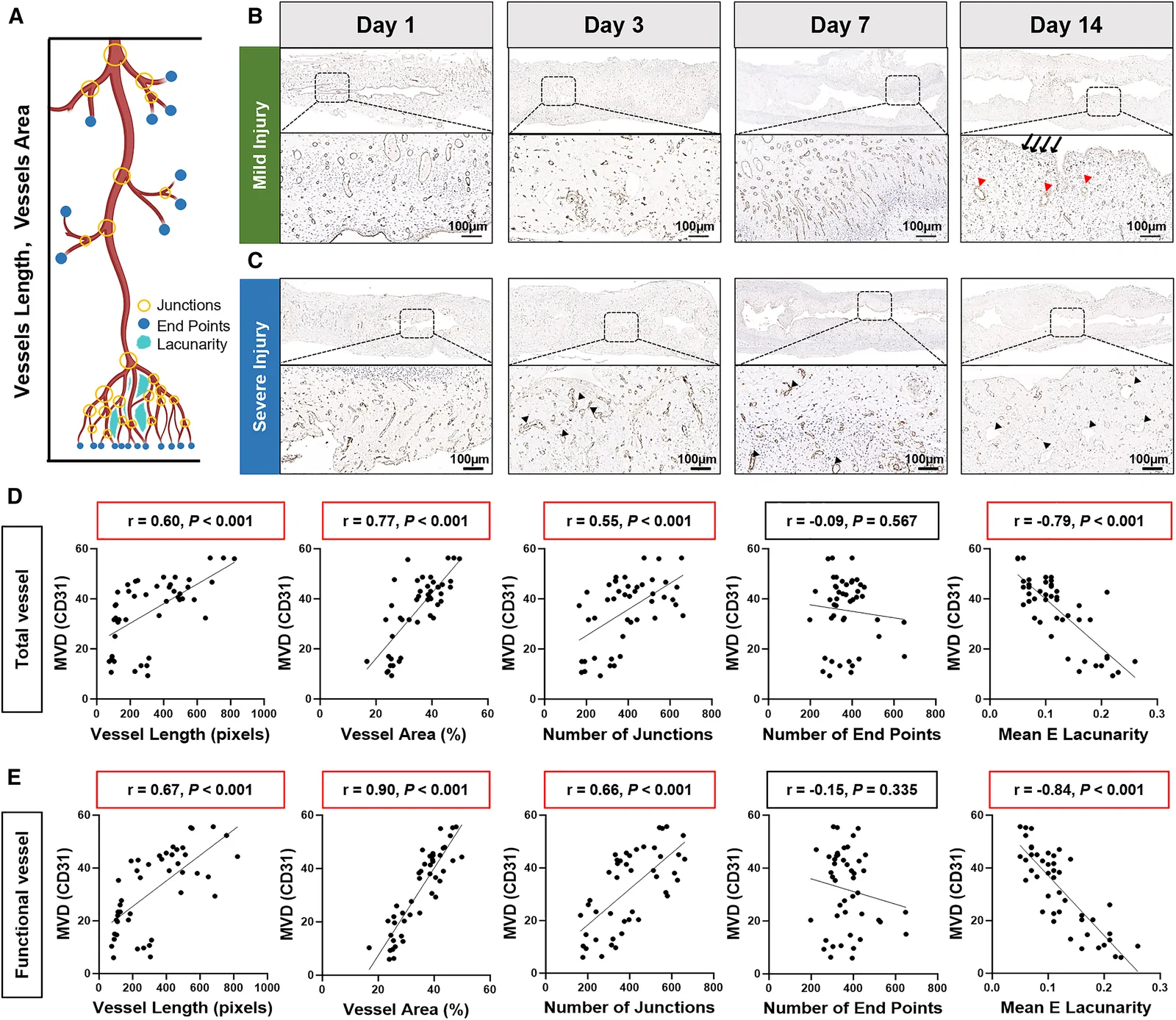
Quantitative analysis of CD31 staining and its correlation with intrauterine PAE-related vascular features (A) Schematic overview of the quantitative PAE imaging features assessed. (B and C) CD31 immunohistochemical staining results in the postinjury endometrium at different time points in the mild and severe injury groups. Red arrows indicate endometrial glands; black short arrows indicate injured vessels; and black long arrows indicate regenerating endometrial epithelial cells. (D and E) Correlation analyses between PAE imaging features and MVD quantified from CD31-stained histological sections, shown for the total vessel set (D) and the functional vessel set (E) Compared with total vessels, PAE features correlated more closely with functional vessels. *n* = 45 biological replicates. Scale bars, 100 μm.

**Table 2:** Histopathologic indexes in different groups

Variable	Normal	Injury severity	Day 1	Day 3	Day 7	Day 14
CD31-MVD (functional vessels)	43.73 ± 3.00	Mild	52.53 ± 4.77^a^	22.87 ± 3.17^a^^,^^b^	40.27 ± 3.50^b^^,^^c^	44.87 ± 3.20^b^^,^^c^
		Severe	35.60 ± 5.71^a^	25.27 ± 5.99^a^^,^^b^	12.40 ± 2.53^a^^,^^b^^,^^c^	8.27 ± 3.04^a^^,^^b^
Inflammatory cells/HPF	2.80 ± 0.38	Mild	5.40 ± 1.61^a^	10.07 ± 2.33^a^^,^^b^	7.80 ± 3.28^a^	4.67 ± 1.18^a^^,^^c^
		Severe	7.93 ± 1.94^a^	15.20 ± 5.24^a^^,^^b^	21.33 ± 3.86^a^^,^^b^	22.53 ± 6.55^a^^,^^b^^,^^d^
Positive staining, Masson (%)	1.18 ± 0.14	Mild	2.85 ± 1.54	4.22 ± 1.09^a^	7.59 ± 3.76^a^^,^^b^	3.41 ± 0.63^a^^,^^d^
		Severe	42.00 ± 5.73^a^	42.82 ± 7.18^a^	45.21 ± 10.46^a^	35.38 ± 4.34^a^^,^^d^
Positive staining, α-SMA (%)	0.07 ± 0.03	Mild	0.45 ± 0.12^a^	0.46 ± 0.17^a^	0.67 ± 0.11^a^^,^^b^	0.51 ± 0.07^a^
		Severe	7.68 ± 1.94^a^	9.51 ± 1.56^a^	7.22 ± 1.21^a^	7.62 ± 1.66^a^

MVD: microvessel density; data are presented as the means ± SDs. Statistical differences between groups were assessed with one-way ANOVA, *n* = 5 biological replicates.

a *p* < 0.05 versus the control group.

b *p* < 0.05 versus the 1-day group.

c *p* < 0.05 versus the 3-day group.

d *p* < 0.05 versus the 7-day group.

Notably, high-power microscopy on day 3 revealed numerous abnormal vessels with irregularly expanded lumina and swollen, discontinuous endothelial cells in both groups (Figure S2). These abnormal vessels were rarely observed in the mild injury group by days 7–14, but persisted in the severe injury group. To further assess the role of PAE in visualizing functional vasculature, we analyzed the correlation between PAE imaging features and the MVD both with (Figure 4D) and without (Figure 4E) abnormal vessels. The correlations between PAE features and MVD improved after excluding abnormal vessels: vessel length (Pearson’s r = 0.67, 95% CI: 0.47–0.81 vs. 0.60, 0.37–0.76), vessel area (r = 0.90, 95% CI: 0.82–0.94 vs. 0.77, 95% CI: 0.62–0.87), number of junctions (r = 0.66, 95% CI: 0.46–0.80 vs. 0.55, 95% CI: 0.30–0.72), and mean E lacunarity (r = −0.84, 95% CI: −0.91–0.72 vs. −0.79, 95% CI:−0.88–0.65). This observation suggests that PAE signals from structurally normal vascular regions are strongly associated with histologically validated vascular density, supporting the potential utility of these signals for assessing angiogenesis-related microenvironmental alterations.

### Detection of endometrial microvascular dynamics by PAE and analysis of its correlation with inflammatory burden

Given that persistent inflammation contributes to endometrial fibrosis, we examined the correlation between PAE features and histological inflammatory burden. The inflammatory burden was defined as the average count of inflammatory cells (infiltrating leukocytes) per high-power field (HPF) on H&E-stained sections, encompassing neutrophils, lymphocytes, plasma cells, and macrophages within the endometrial stroma. H&E staining revealed a significant increase in inflammatory cells/HPF in both injury groups on day 3 postinjury. In the mild injury group, inflammatory cell counts peaked on day 3, decreased by day 7, and returned to near-normal levels by day 14. The infiltrates at this stage primarily consisted of chronic inflammatory cells, such as plasma cells and lymphocytes (Figures 5A and 5B and Table 2). In contrast, the severe injury group maintained a high inflammatory burden through days 7–14 (Figure 5C), with a detectable proportion of acute inflammatory cells (e.g., neutrophils) still present (Figure 5A). A correlation heatmap revealed associations between PAE-derived features and inflammatory cell density from day 7 onward. Notably, on day 7, the vessel area (Pearson’s r = −0.91, 95% CI: −0.98 to −0.67), number of junctions (r = −0.92, 95% CI: −0.98 to −0.70), and mean E lacunarity (r = 0.86, 95% CI: 0.49 to 0.97) were significantly correlated with the inflammatory burden (Figures 5D and S3). These findings indicate that PAE-derived microvascular features are correlated with the inflammatory burden in the postinjury microenvironment.

**Figure 5:**
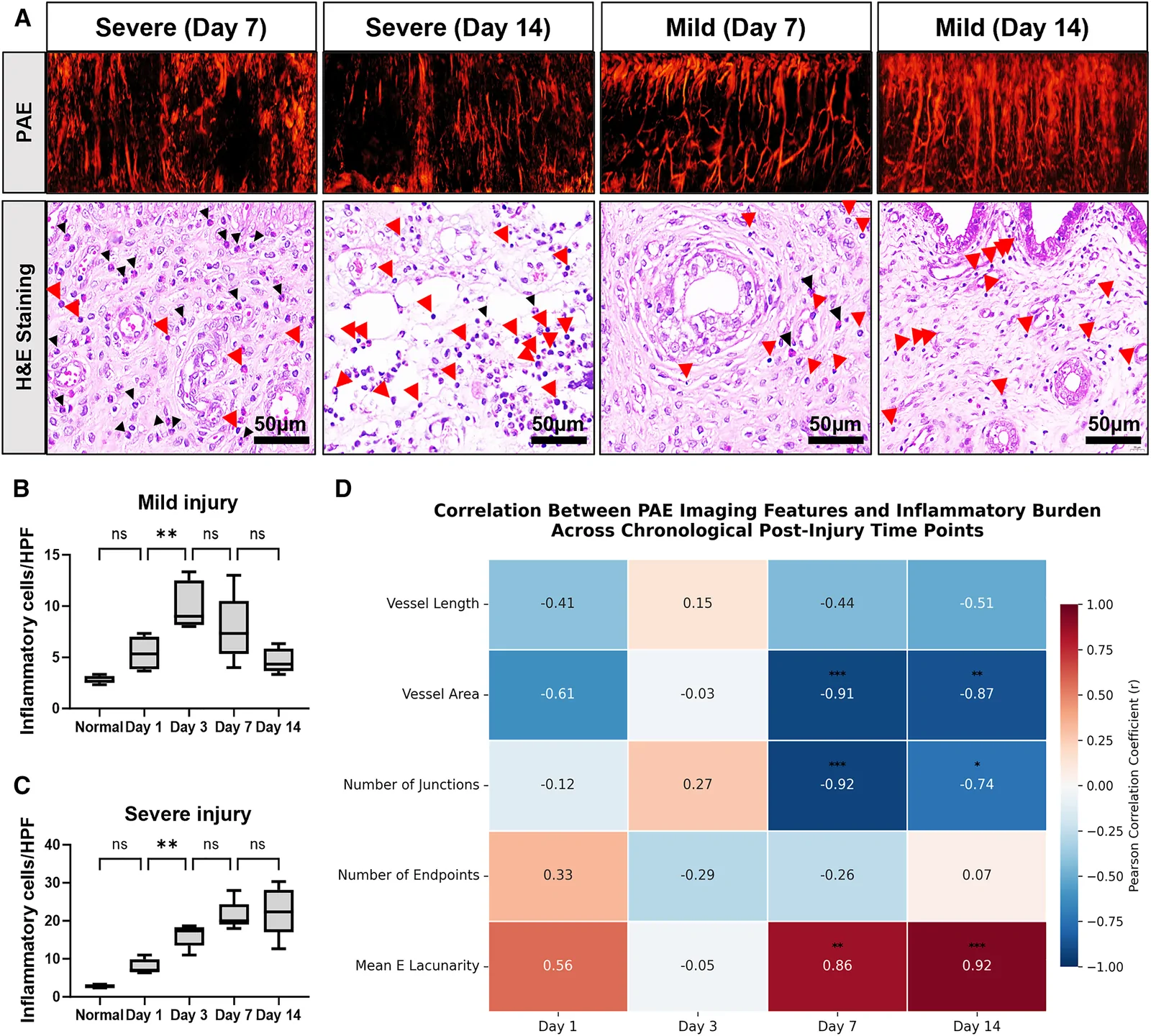
Histopathological quantification of the inflammatory burden and its correlation with intrauterine PAE imaging features (A) Representative H&E-stained sections and corresponding intrauterine PAE images at 7 and 14 days postinjury. Red arrows indicate chronic inflammatory cells (e.g., plasma cells and lymphocytes); black arrows indicate acute inflammatory cells (e.g., neutrophils). (B and C) Quantitative analysis of inflammatory cells/HPF in the mild injury group (B) and the severe injury group (C). (D) Correlation heatmap between quantitative PAE-derived features and inflammatory cells/HPF across different postinjury stages. The data are presented as mean ± SD, *n* = 5 biological replicates. ∗*p* < 0.05, ∗∗*p* < 0.01, ∗∗∗*p* < 0.001 by two-way ANOVA followed by Tukey’s post hoc test; ns, not significant. Scale bars, 50 μm.

### Early evaluation of endometrial fibrosis based on intrauterine PAE imaging features

To quantitatively assess the performance of PAE in the early evaluation of endometrial fibrosis, we analyzed the correlations between PAE imaging features and established histopathological indicators of fibrosis. These key PAE features were subsequently used for early evaluation of postinjury endometrial fibrosis. We evaluated two pathological fibrosis metrics, namely, the collagen area (by Masson’s trichrome staining), which represents terminal matrix deposition, and myofibroblast enrichment (by α-SMA IHC), which represents active fibrotic activity. Spearman correlation analysis revealed a strong correlation between the percentage of Masson-stained collagen area and the α-SMA-positive area ratio across all samples (r = 0.87, *p* < 0.001, Figure S4). Finally, collagen proportionate area (CPA) measured on day 14 by Masson staining was selected as the primary histological endpoint for fibrosis. Compared with normal and mild injury, severe injury resulted in significantly increased fibrosis (blue-stained area) (Figure 6A). No significant differences in fibrosis levels were observed across the evaluated time points within the mild or severe injury group (Figures 6B and 6C), indicating that the fibrosis model was stable over time. A correlation heatmap revealed associations between multiple PAE-derived features and fibrosis outcome (Figure 6D). The correlations between PAE features and fibrosis outcomes were generally weak and nonsignificant on day 1 and day 3 (average |r| = 0.407 and 0.156, respectively) but became substantially stronger at later stages. Consistent with the “microperfusion-inflammation-fibrosis” cascade following endometrial injury, the strongest overall correlations were observed on day 7 postinjury (average |r| = 0.679). At this time point, three key features showed significant associations with fibrosis outcome: vessel area (Pearson’s r = −0.76, 95% CI: −0.94 to −0.26), number of junctions (r = −0.90, 95% CI: −0.98 to −0.62), and mean E lacunarity (r = 0.90, 95% CI: 0.61 to 0.98). The complete correlation matrices for all timepoints are provided in Figure S5. On the basis of the results of the integrative analysis, we inferred that day 7 postinjury represents the optimal time point for evaluating endometrial fibrosis using key PAE-derived features (vessel area, number of junctions, and mean E lacunarity).

**Figure 6:**
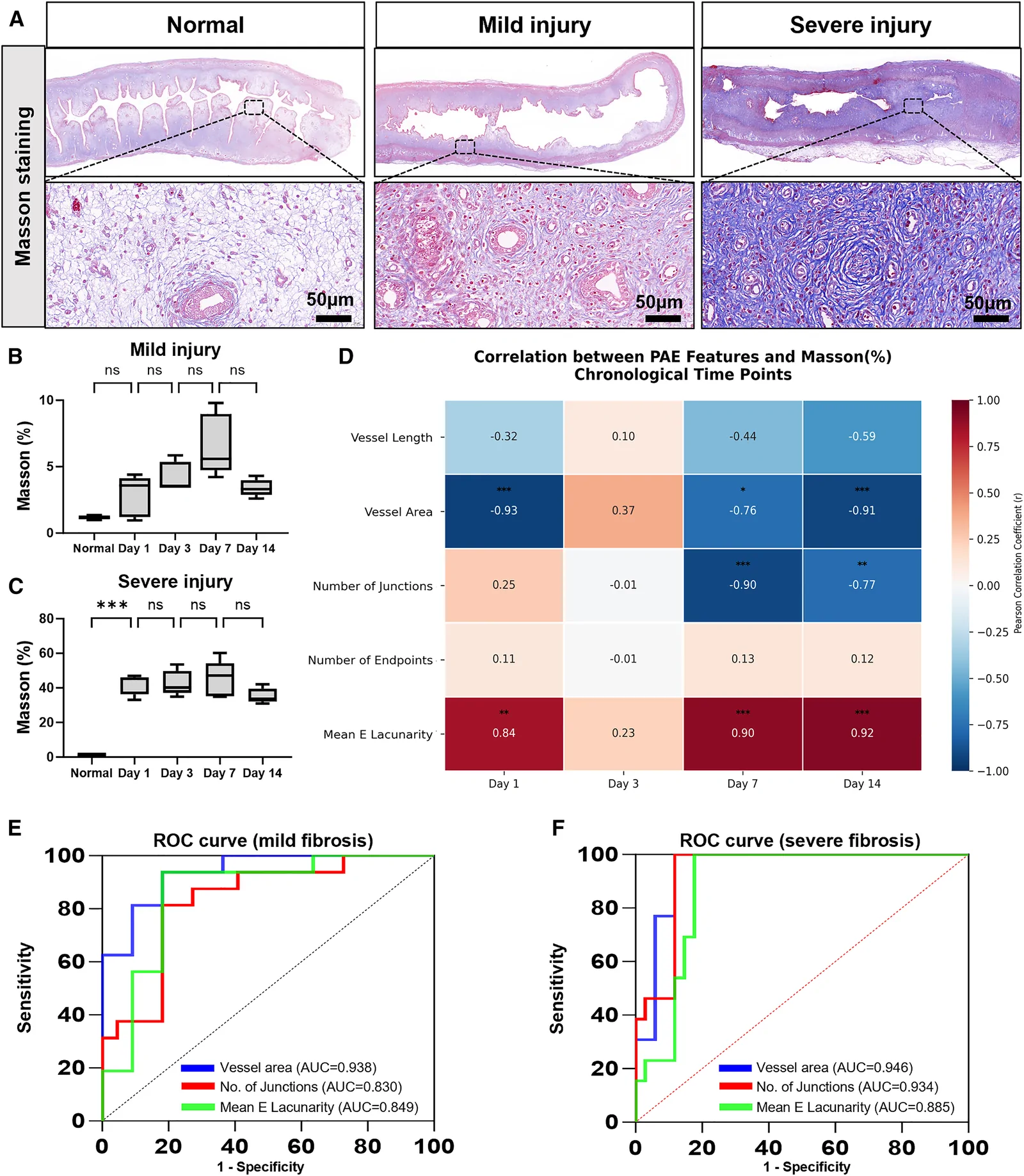
Quantitative analysis of PAE-derived features and their correlation with fibrosis (A) Representative Masson’s trichrome-stained sections from different injury severity groups. (B and C) Quantification of the CPA in the mild injury group (B) and the severe injury group (C), *n* = 5 biological replicates. (D) Correlation heatmap between quantitative PAE imaging features and the CPA across various postinjury stages. (E and F) ROC analysis for distinguishing mild (E) and severe (F) fibrosis based on PAE imaging features, *n* = 30 biological replicates. The data are presented as mean ± SD. ∗*p* < 0.05, ∗∗*p* < 0.01, ∗∗∗*p* < 0.001 by two-way ANOVA followed by Tukey’s post hoc test; ns, not significant. Scale bars, 50 μm.

Fibrosis severity grading was defined by the quantitative CPA. The grading threshold was set at the mean plus two standard deviations of the CPA in the control and injury groups in the study cohort. Using this benchmark, we established the following boundaries: 1.5% as the upper limit for the normal endometrium, 10% as the cutoff between normal tissue and mild fibrosis, and 30% as the threshold distinguishing mild from severe fibrosis (Table 3). The 30% threshold for distinguishing severe from mild fibrosis was determined based on the distribution of CPA values across the mild and severe injury groups. This threshold was further supported by histopathological evaluation, which consistently identified dense collagen deposition with marked glandular destruction in sections with CPA ≥30%, whereas sections below 30% showed milder structural alterations. The performance of the three PAE imaging features measured on day 7 was subsequently evaluated by receiver operating characteristic (ROC) curve analysis using an independent validation dataset of 30 samples. All confidence intervals were estimated using bootstrapping with 1,000 resamples. For the detection of severe fibrosis (CPA ≥30%), the AUCs were 0.946 for vessel area (95% CI: 0.860–1.000), 0.934 for the number of junctions (95% CI: 0.841–1.000), and 0.885 for mean E lacunarity (95% CI: 0.753–1.000) (Figure 6E). For discriminating between normal tissue and mild fibrosis (CPA <10%), the corresponding AUCs were 0.938 (95% CI: 0.850–1.000), 0.830 (95% CI: 0.664–0.996), and 0.849 (95% CI: 0.682–1.000), respectively (Figure 6F). The cutoff value, sensitivity, specificity, and accuracy are listed in Table 4.

**Table 3:** Quantification of collagen area percentage (by Masson’s trichrome staining) across injury severity groups and definition of fibrosis severity grading

Groups	Mean (%)	SD (%)	Mean + 2SD
Normal group	1.178	0.144	1.466
Mild injury group	4.518	2.715	9.948
Severe injury group	40.353	9.377	59.107
**Fibrosis severity grading**	Mild fibrosis	1.466–9.948	
	Severe fibrosis	9.948–30.287∗	

SD: standard deviation. ∗30.287 ≈ (59.107 + 1.466)/2.

**Table 4:** ROC curve analysis of PAE imaging features in early evaluation of endometrial fibrosis

PAE features	AUC value	95%CI	Cutoff value	Sensitivity	Specificity	Accuracy
Vessel area-MF (%)	0.938	0.850–1.000	29.78	0.909	0.812	0.926
No. of junctions-MF	0.830	0.664–0.996	280.50	0.818	0.812	0.889
Mean E lacunarity-MF	0.849	0.682–1.000	0.125	0.938	0.818	0.889
Vessel area-SF (%)	0.946	0.860–1.000	30.05	0.882	1.000	0.949
No. of junctions-SF	0.934	0.841–1.000	280.50	0.882	1.000	0.949
Mean E lacunarity-SF	0.885	0.753–1.000	0.135	1.000	0.824	0.900

MF: mild fibrosis, SF: severe fibrosis.

## Discussion

In this study, we established an intrauterine PAE-based method for spatiotemporal monitoring of the post-injury endometrial microenvironment to investigate the association between early imaging features and subsequent fibrosis progression. PAE imaging features correlated with dynamic changes in endometrial microvascular networks and inflammatory burden, indicating the ability of this method to detect alterations in microperfusion and inflammatory activity. This approach enables the identification of injury severity at both early and late stages and facilitates early assessment of fibrosis progression. PAE-derived parameters, including vessel length, number of junctions, and mean E lacunarity, were significantly associated with histopathological microvascular and inflammatory outcomes, and may serve as useful indicators for evaluating mild or severe fibrosis from day 7 images. No laser-related tissue damage was observed in any animal, supporting the safety of the procedure for potential *in vivo* applications. Together, these findings suggest that intrauterine PAE offers a direct, high-resolution imaging method for monitoring the endometrial microenvironment, which may contribute to early evaluation of fibrosis and inform strategies for early intervention.

The outcome of endometrial injury is determined by a critical balance between the tissue’s intrinsic repair capacity and the extent of injury—a balance modulated by factors such as microvascular dynamics, chronic injury, infection status, and other unknowns.^1^^,^^22^ Evidence indicates that microvascular damage is closely related to fibrosis in IUA, characterized by reduced expression of VEGF and CD34, potentially mediated through pathways such as hypoxia- or fibrosis-related signaling (e.g., the Notch-Smad and HIF-1α-VEGF axes).^23^ Additionally, angiogenesis is essential for endometrial repair, as it activates endogenous regenerative mechanisms, enhances receptivity, and ensures adequate nutrient and oxygen supply to the injured site.^24^ Although dynamic monitoring of microperfusion is important for understanding IUA formation and developing early interventions, it remains technically challenging. Conventional Doppler ultrasound, for example, cannot reliably resolve vessels less than 1 mm in diameter, limiting its utility for tracking microvascular dynamics.^25^ In this study, using advanced PAE imaging at micrometer resolution, we tracked microvascular changes postinjury.^26^ The PAE variables revealed distinct patterns between the mild and severe injury groups. In the early phase (day 1–3), the vessel area and length were significantly lower in the severe injury group, which correlated with the severity of microvascular injury. The later-phase (day 7–14) metrics suggested impaired vascular repair in the severe injury group. This was evidenced by a widening disparity in vessel area, length, and number of junctions (reflecting angiogenesis capacity), coupled with a significantly greater mean E lacunarity (negatively correlated with vascular density) than in the mild injury group.

The interplay between angiogenesis and inflammation plays a key role in both fibrosis development and functional repair after injury.^1^ For example, a previous study indicated that chronic endometritis was associated with reduced endometrial MVD, which was attributed to an inflammatory-induced deficit in sphingosine-1-phosphate signaling that compromises vascular remodeling and angiogenesis.^27^ In our experiment, the inflammatory response in the mild injury group peaked on day 3 postinjury before gradually declining, which is consistent with prior pathological evidence that leukocyte recruitment occurred within the first two days.^28^ On day 3 postinjury, concurrent pathological findings revealed widespread inflammation, tissue edema, and microvascular injury. The ensuing vascular dysfunction included erythrocyte extravasation, a decrease in lamina cells, endothelial damage, and focal necrosis. The corresponding PAE findings featured typical blood flow stasis and decreased vascular density, with a higher mean E lacunarity, reflecting increased heterogeneity.^29^ PAE parameters correlated more strongly with functional microvessels than with total micro-vessel count, highlighting their potential for assessing functional vasculature. The gradual return of microvascular parameters to normal over time in the mild injury group demonstrated its capacity for self-repair, which was consistent with the vascular regeneration time observed by PAE in previous gastrointestinal injury models.^19^ Conversely, severe injury precipitated a cascading pathological process. Histopathology revealed persistent acute inflammation (e.g., neutrophils), while PAE imaging revealed a progressive loss of microvascular signals, with both alterations persisting from day 7 through day 14.

In the clinical assessment of endometrial fibrosis or IUA, conventional diagnostic methods—such as hysteroscopy and biopsy—primarily detect pathology at an advanced stage, making early intervention challenging. Although clinical factors (e.g., injury severity and concurrent endometritis), ultrasound features, and platelet parameters have been explored as potential indicators,^30^^,^^31^^,^^32^ their ability to provide direct, noninvasive, and serial visualization of disease progression remains limited. By integrating three-dimensional spatial mapping with temporal observation, PAE enables the simultaneous assessment of lesion location and progression over time. This combined spatiotemporal information may provide a potential approach for the early evaluation of fibrosis outcomes in IUA patients. On the basis of the correlation between quantitative PAE features and histopathological fibrosis, we identified day 7 as a key time point for early evaluation of fibrosis using PAE imaging, a finding that aligns with the underlying pathophysiology of endometrial fibrosis. At this time point, the acute inflammatory response initiated by injury begins to subside, while inadequate microvascular repair becomes increasingly apparent—features that characterize the transition toward fibrotic progression.^28^ To establish thresholds for fibrosis staging, we quantified pathological collagen fibers and correlated these with PAE features. ROC analysis demonstrated the diagnostic performance of the PAE-derived vessel area, number of junctions, and mean E lacunarity, supporting their potential for early evaluation of fibrosis, particularly in severe cases.

In conclusion, our findings indicate that quantitative PAE, which captures the spatiotemporal dynamics of the postinjury microvascular and inflammatory microenvironment, may offer a useful approach for the early evaluation of endometrial fibrosis. This study identifies a set of serially quantifiable, PAE-based microvascular morphological parameters that could serve as imaging biomarkers for characterizing the endometrial injury repair process, potentially informing future strategies for the clinical management of endometrial injury and fibrosis.

### Limitations of the study

While this study provides insights into the potential of PAE for monitoring the postinjury endometrial microenvironment and for the early evaluation of fibrosis, several limitations should be acknowledged. First, although our results indicate that early-phase PAE signal enhancement followed by vascular loss is consistent with inflammation-driven angiogenesis and subsequent fibrosis, the underlying mechanisms warrant further investigation. A deeper understanding of the relationship between PAE imaging features and the molecular- and protein-level mechanisms of tissue repair and vascular remodeling would provide more substantial evidence to support the utility of PAE for monitoring endometrial changes. Second, compared with the imaging biomarkers of endometrial damage identified in optical coherence tomography (OCT) studies,^33^^,^^34^ PAE focuses more specifically on providing vascular structural and dynamic functional information. The integration of structural imaging modalities, such as OCT or ultrasound, would facilitate a more comprehensive understanding of the microenvironmental changes following endometrial injury. Third, the use of a single wavelength (532 nm) does not permit differentiation between signals from perfused microvessels and those from non-perfused hemoglobin pools (e.g., blood clots or congested regions). Accordingly, the early PAE signal enhancement observed should be interpreted as a composite readout of total hemoglobin content and tissue hyperemia, rather than as a direct measure of functional perfusion alone. Finally, clinical translation of intrauterine PAE requires probe redesign for the larger uterus to ensure adequate depth and resolution, along with consideration of cycle-related thickness changes and systematic safety evaluation. Moreover, the combination of multi-wavelength PAI (e.g., collagen imaging) or multimodal imaging (e.g., with ultrasound) may offer improved assessment across multiple dimensions. Our future work will focus on large animal models (e.g., pigs) and pilot clinical studies to further validate the clinical applicability of PAE. Despite these limitations, we believe that demonstrating the feasibility of PAE may encourage further investigation into endometrial microperfusion dynamics and support its potential for early evaluation of endometrial fibrosis.

## Resource availability

### Lead contact

Further information and requests for resources and reagents should be directed to and will be fulfilled by the lead contact, Zhiyi Chen (zhiyi_chen@usc.edu.cn).

### Materials availability

This study did not generate new unique reagents.

### Data and code availability

• Data reported in this study will be shared by the lead contact upon request.
• This article does not report original code.
• Any additional information required to reanalyze the data reported in this article is available from the lead contact upon request.

## Acknowledgments

This study was supported by 10.13039/501100001809National Natural Science Foundation of China (no. 82102054, U22A2034), the Postdoctoral Fellowship Program of CPSF (no. GZC20261178), Clinical Research 4310 Program of the Affiliated Changsha Central Hospital of the 10.13039/501100009020University of South China (no. 20214310NHYCG06), Health Research Project of Hunan Provincial Health Commission (no. W20241010), Major Program from Xiangjiang Laboratory (no. 23XJ02005), Key Research and Development Programs of 10.13039/501100002767Department of Science and Technology of Hunan Province (no. 2024JK2135), the Scientific Research Fund of Hunan Provincial Education Department (no. 24A0018) and 10.13039/501100002822Central South University Research Program of Advanced Interdisciplinary Studies (no. 2023QYJC020).

## Author contributions

Conceptualization, X.L. and Z.C.; methodology, X.L., Z.G., K.X., Z.H., D.Z., S.K., H.C., M.D., X.K., and X.W.; investigation, X.L. and Z.G.; writing – original draft, X.L.; writing – review and editing, Z.G., X.W., and Z.C.; funding acquisition, X.K. and Z.C.; resources, X.K., X.W., and Z.C.; supervision, M.D., X.W., and Z.C.

## Declaration of interests

The authors declare no competing interests.

## STAR★Methods

### Key resources table

**Table:** 

REAGENT or RESOURCE	SOURCE	IDENTIFIER
**Antibodies**		

CD31		RRID: AB182981
α-SMA		RRID: AWA10574

**Critical commercial assays**		

PAE system (ANI-PAE10)	Guangdong Photoacoustic Technology Co., Ltd.	http://www.pamedical.com.cn/gsdmtnkj

**Software and algorithms**		

Angiotool	National Cancer Institute	https://ccrod.cancer.gov/wiki-html/ROB2/Home_62196320.html
ImageJ	National Institute of Health	https://imagej.nih.gov/ij/
SPSS	IBM	https://www.ibm.com/products/spss
Python	Python software	https://www.python.org
GraphPad Prism	GraphPad Software	https://www.graphpad.com/

### Experimental model and study participant details

#### Animals

Female Sprague–Dawley rats (8–10 weeks old) were obtained from Silaikejingda Experimental Animal Co., Ltd. (Hunan, China). The rats were kept under controlled conditions at a temperature of 24 ± 2°C, a 12-hour light/dark cycle, and a relative humidity maintained at 50 ± 5%. The study was approved by the Animal Ethics Committee of Hunan Prevention and Treatment Institute for Occupational Diseases (Approval No. HNZFY-2025-011) and followed the ARRIVE guidelines.

### Method details

#### Study design and dataset

A total of 45 rats were allocated to three groups: a sham-operated control group (*n* = 5), a mild injury group (*n* = 20), and a severe injury group (*n* = 20). Animals in the mild and severe injury groups were further divided into four subgroups (*n* = 5) corresponding to the postinjury time points of 1, 3, 7, and 14 days. The rats in the estrous phase (confirmed by vaginal cytology via Wright‒Giemsa staining) underwent laparotomy under anesthesia for endometrial fibrosis modeling. An ethanol-induced rat model, which has been widely used to study endometrial fibrosis,^35^ was used in this study. To simultaneously dynamic monitoring of the achieve *in vivo* microenvironment and endpoint histologic validation, we employed a bilateral uterine-cavity modeling design in rats. Standardized injury was induced in both uterine horns of each rat by perfusion with 95% ethanol. The duration of perfusion was 30 s in the mild injury group and 90 s in the severe group. The uterine cavity was then thoroughly lavaged with saline. The surgical incisions were closed with absorbable sutures, followed by postoperative analgesia and small-dose antibiotic prophylaxis. All groups underwent the same surgical procedures to ensure valid intergroup comparisons. In addition, an independent validation set of 30 female rats (mild injury: severe injury = 1:1) was established. The study design, PAE image processing and animal allocation are illustrated in Figure S6.

#### PAE system and imaging protocol

The PAE imaging system employed an ultrathin photoacoustic microcatheter endoscope consisting of a miniaturized lensed fiber-optic capsule, ultrasonic transducer, and torque coil for rotational scanning (diameter: 1.4 mm). Using a helical pullback scanning protocol, the PAE probe acquired cross-sectional circular B-scan images at a frame rate of 20 frames per second while being withdrawn along the longitudinal axis at 0.4 mm/s. This approach enabled a 3D photoacoustic scan over a 30 mm length of the uterine cavity to be completed within 75 seconds, providing comprehensive volumetric data on microvascular network architecture with a maximum imaging depth of 1.5 mm. The single-wavelength PAE system operated at 532 nm, delivering an optical lateral resolution of approximately 16 μm. The system was constructed based on our previously reported design.^18^ Schematics of the system and the probe are shown in Figure 1.

For animal *in vivo* imaging, at 1, 3, 7, and 14 days after modeling, the PAE probe with an outer sheath was inserted into one side of the uterus through the incision conducted on the uterus near the ovary side for continuous rotational scanning from the proximal vaginal end to the proximal ovarian end. The outer sheath was filled with water to achieve acoustic coupling. Once positioned, the PAE imaging procedure was executed. The imaging side of the uterine cavity and its supplying blood vessels were immediately ligated, and the uterine tissues were collected after PAE imaging. The raw PAE imaging data were subsequently imported into MATLAB R2021b (MathWorks) to reconstruct the endometrial microvascular structure and perform MAP, DEVM, and 3D reconstruction mapping. To ensure technical reproducibility, all the imaging procedures were performed following a strict standard operating procedure. This included (1) consistent positioning of the animal and the endoscopic probe and (2) fixed imaging parameters (e.g., laser fluence, detector gain, and scanning speed) for all acquisitions.

#### Image processing and analysis

The original PAE images were cropped into two separate regions of interest (ROIs), with the central vascular confluence area excluded to avoid regions extending beyond the endometrial cavity. AngioTool (Ver. 0.6a, NIH)^36^ was used to quantify the vascular features in each ROI independently, and the average values of the two ROIs were calculated as the final PAE metrics for each rat. The extracted parameters included the percentage of vessel area (hereafter vessel area), average vessel length (hereafter vessel length), number of junctions, number of end points, and mean E lacunarity. We adjusted the segmentation parameters to identify vascular structures (defined as tubular or branching signals) within each ROI. Regions exhibiting clustered, plaque-like, or diffuse photoacoustic signals suggestive of congestion or hemorrhage were preferentially excluded where feasible. To assess the consistency and reproducibility of these features, two experienced radiologists independently performed vessel delineation and feature extraction. The intraclass correlation coefficient (ICC) was computed to assess interobserver agreement; only features with an ICC ≥ 0.75 were retained for subsequent analysis, ensuring that the data were robust and reliable. The ICCs and 95% CIs of the PAE features are presented in Table S1.

#### Histopathology procedure

Histopathological analysis was performed to validate the ability of PAE to characterize endometrial microvessels at different stages after injury and to assess fibrosis. The uterine tissue from the imaged side was immediately collected and fixed in 4% aldehyde fixative after imaging. Hematoxylin‒eosin staining was subsequently performed to determine inflammatory cell counts for histological analyses of inflammatory progression, and immunohistochemical (IHC) staining with anti-CD31 antibodies was performed to assess angiogenesis. The contralateral uterine cavity was preserved *in situ*, and the rat was maintained until sacrifice on postinjury day 14, at which time this side of the uterine cavity was collected to serve as the gold standard for assessing the final fibrosis outcome through Masson staining and IHC staining with anti α-SMA. Fibrosis was quantitatively analyzed by measuring the CPA with ImageJ software. In the absence of a recognized grading standard for endometrial fibrosis in rats, we defined a histopathological grading system based on the method of Qiu et al.^17^ Specifically, the threshold for fibrosis was defined as the mean CPA of the control group plus two standard deviations; this threshold was subsequently applied to classify the severity of fibrosis in the experimental groups.

### Quantification and statistical analysis

Statistical analysis was performed with SPSS software (version 22, IBM) and Python (version 3.10.11). Based on previous studies^21^ and our pilot experiments, a sample size of *n* = 5 per subgroup was estimated to be sufficient to detect significant differences between groups. All the statistical analyses were performed with the animal as the independent analytical unit. The values are presented as means ± SDs for normally distributed data while presented as medians with IQRs for nonnormally distributed data. Normality of data distribution was assessed using the Shapiro–Wilk test, and homogeneity of variances was examined using Levene’s test. For data meeting both assumptions, parametric tests were applied; otherwise, non-parametric equivalents were used. Statistical analyses were performed using two-way ANOVA to assess the effects of injury severity and time post-injury on PAE parameters, followed by Tukey’s post hoc test for multiple comparisons. Relationships between PAE parameters and histologic parameters were assessed using Spearman's rank correlation and are reported as correlation coefficients with corresponding 95% CIs. ROC curves were generated to assess the diagnostic performance in evaluating endometrial fibrosis, and the AUC was calculated as an indicator of the performance of the PAE indicators. Given the sample size, we assessed the stability of the PAE parameter statistics using the bootstrapping method, calculating 95% CIs based on 1000 resampling iterations with replacement. Statistically significant differences among groups are indicated by two-tailed *P* values, with *P* < 0.05 as the threshold. The number of independent replicates and additional statistical details are provided in the figure legends.
