# Supplementary material for: Quantitative photoacoustic endoscopy reveals early microvascular biomarkers of endometrial fibrosis in a rat model
Source: iScience. 2026 Sep 28;29(10):117609. doi: 10.1016/j.isci.2026.117609 (PMC13651823; doi:10.1016/j.isci.2026.117609)
Supplement: Supplementary file 1 [file mmc1.pdf]

iScience, Volume *29*

Supplemental information

Quantitative photoacoustic endoscopy reveals early microvascular biomarkers of endometrial fibrosis in a rat model

Xiaowen Liang, Zhili Guo, Kedi Xiong, Zijian He, Dongyu Zhang, Shihan Kang, HongJi Chen, Meng Du, Xiaoyan Kui, Xue Wen, and Zhiyi Chen

Supplemental file

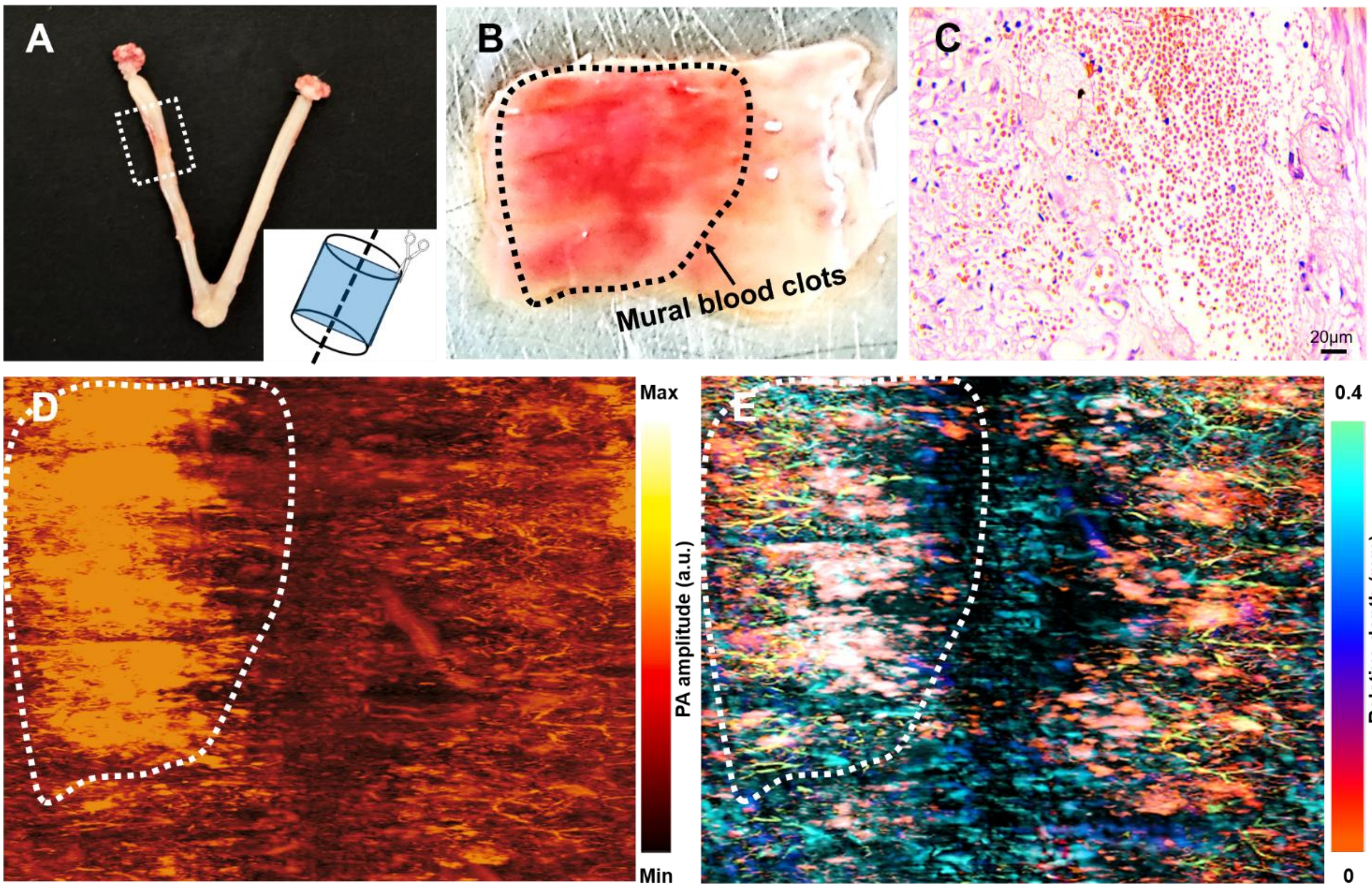

Figure S1. PAE imaging of mural blood clots at day 3 after injury, showing evidence of acute vascular damage with interstitial hemorrhage. (A and B). Gross specimen. (C). Representative H&E-stained section. Scale bar: 20 μm. (D). Representative PAE maximum amplitude projection. E. Representative PAE depth-encoded vascular map.

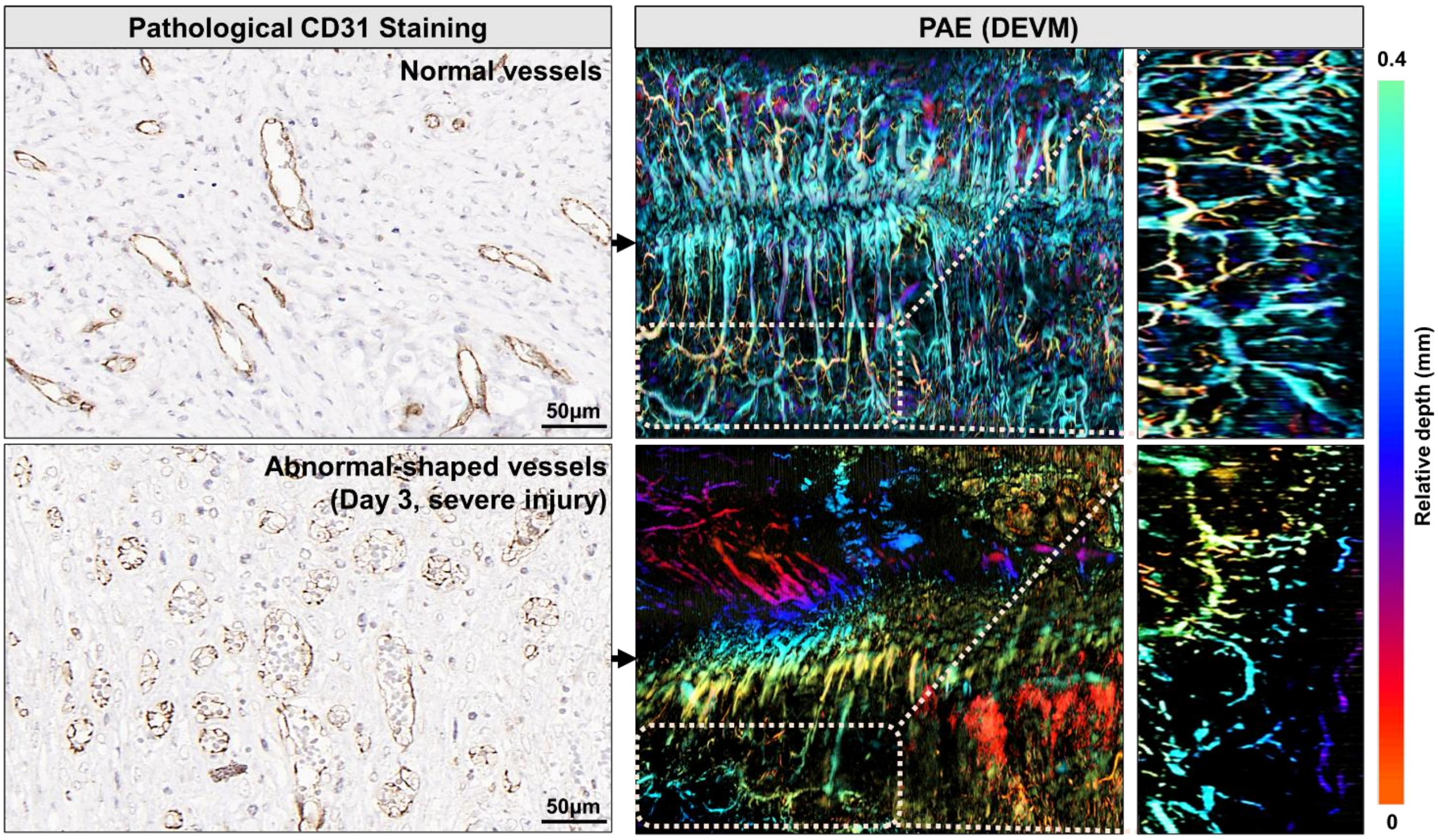

Figure S2. Comparison of normal and abnormally shaped vessels at the pathological and PAE levels. At the pathological level, abnormal vessels exhibit irregularly dilated lumina and discontinuous endothelial lining. In the PAE-DEVM, these vessels demonstrate reduced and discontinuous signals compared with normal vasculature. Scale bar: 50 μm.

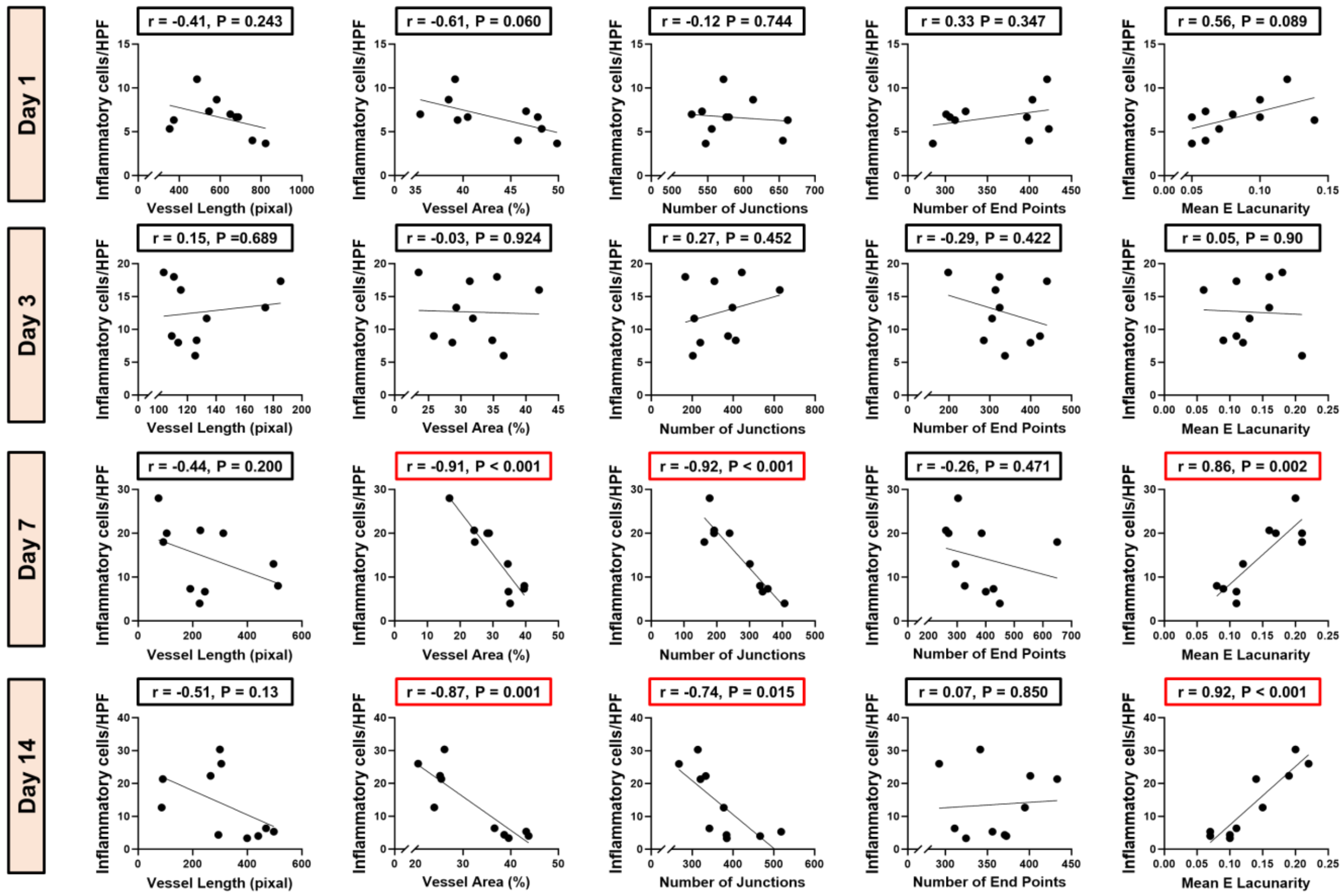

Figure S3. Correlation analysis between quantitative PAE-derived features and inflammatory cells/HPF across different post-injury stages. Data are presented as mean ± SD, n = 10 biological replicates.

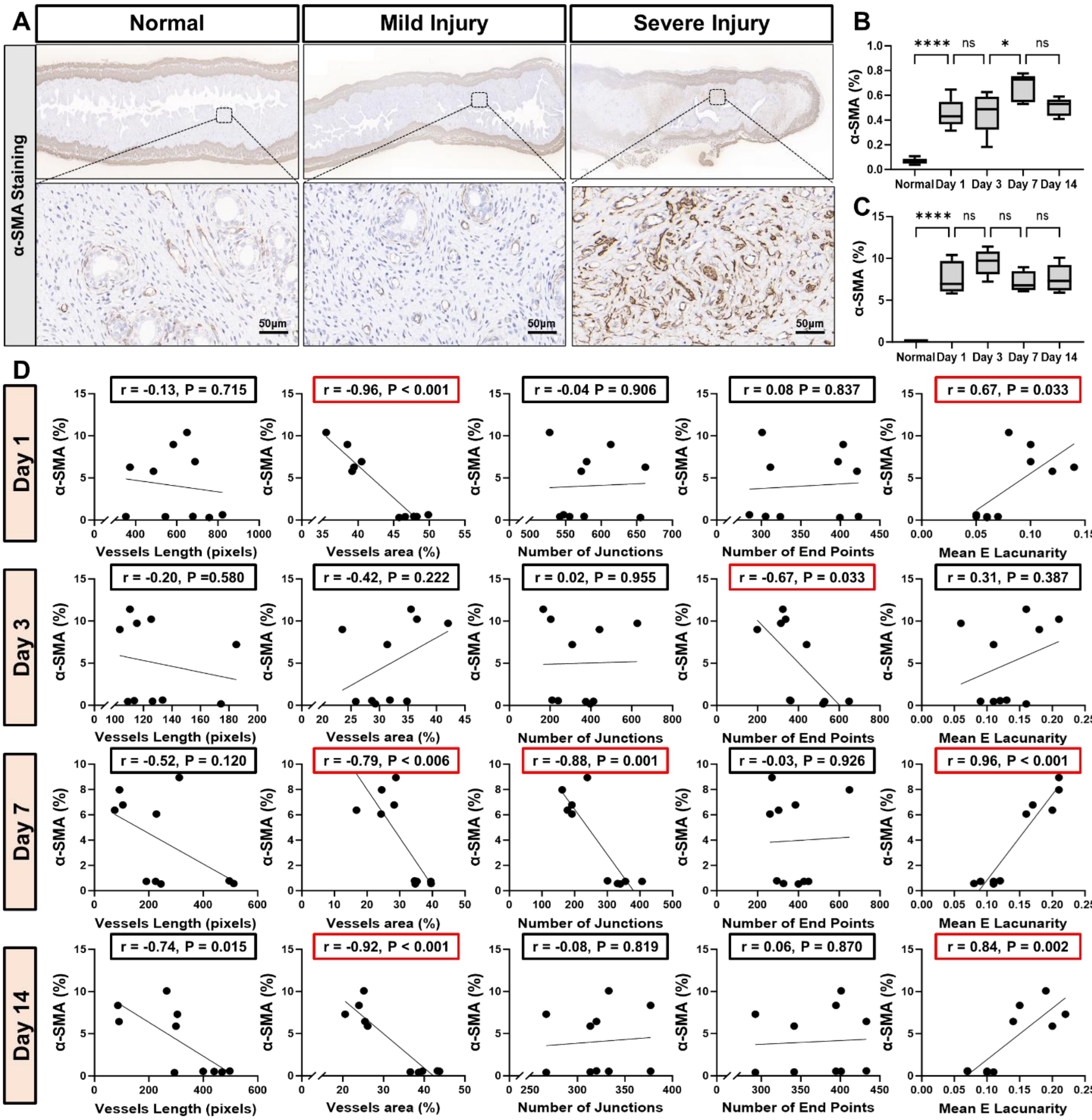

Figure S4. Quantitative analysis of PAE-derived features and their correlation with fibrosis (α-SMA). (A). Representative α-SMA's immunohistochemical staining from different injury severity groups. (B and C). Quantification of the percentage of positive area in the mild injury group (B) and the severe injury group (C), *n* = 5 biological replicates. (D). Correlation between quantitative PAE imaging features and α-SMA positive area across various post-injury stages, *n* = 10 biological replicates. Data are presented as mean ± SD. *P < 0.05; ****P < 0.0001; ns, not significant.

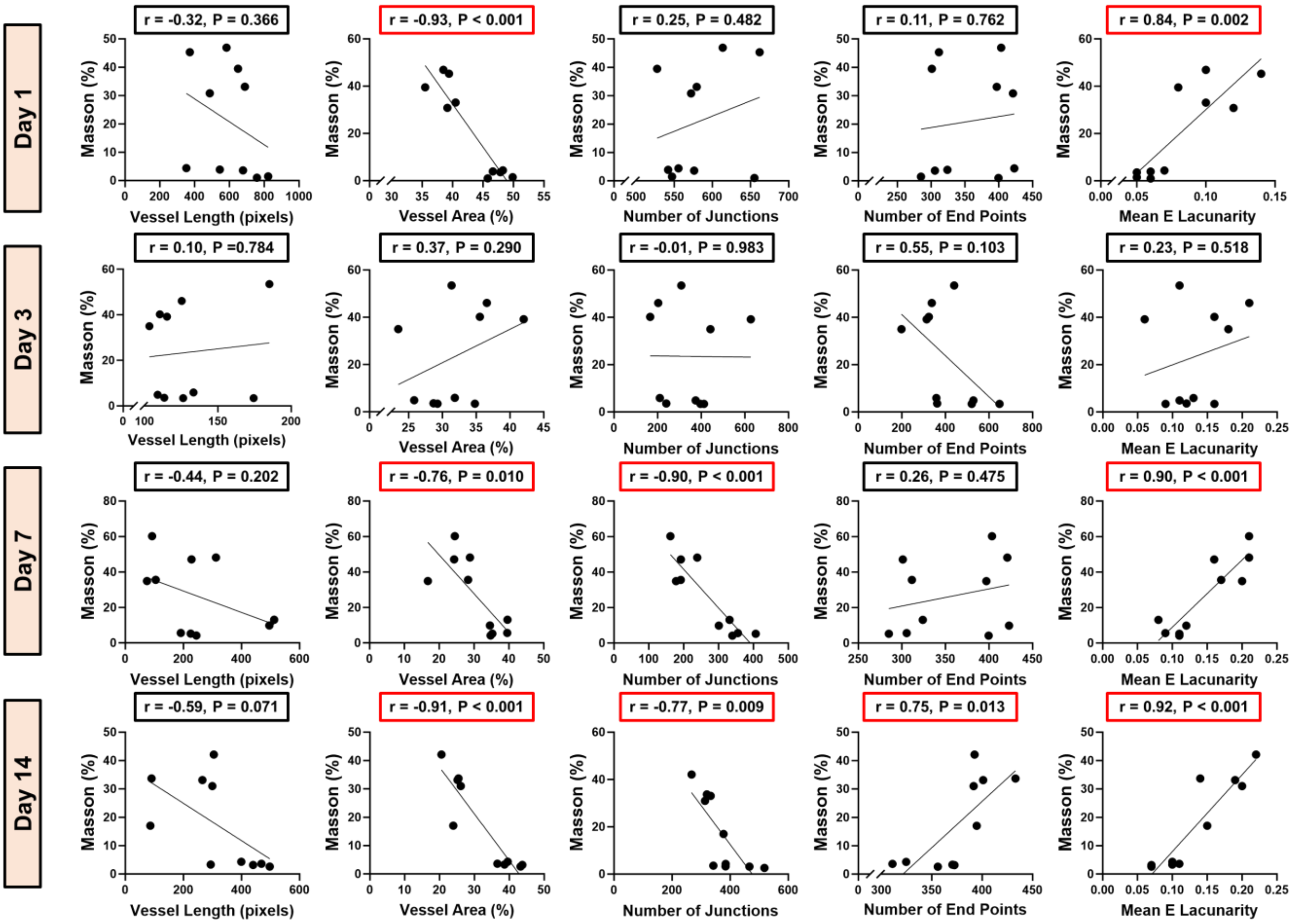

Figure S5. Correlation between quantitative PAE imaging features and the percentage of collagen area across various post-injury stages. Data are presented as mean ± SD, *n* = 10 biological replicates.

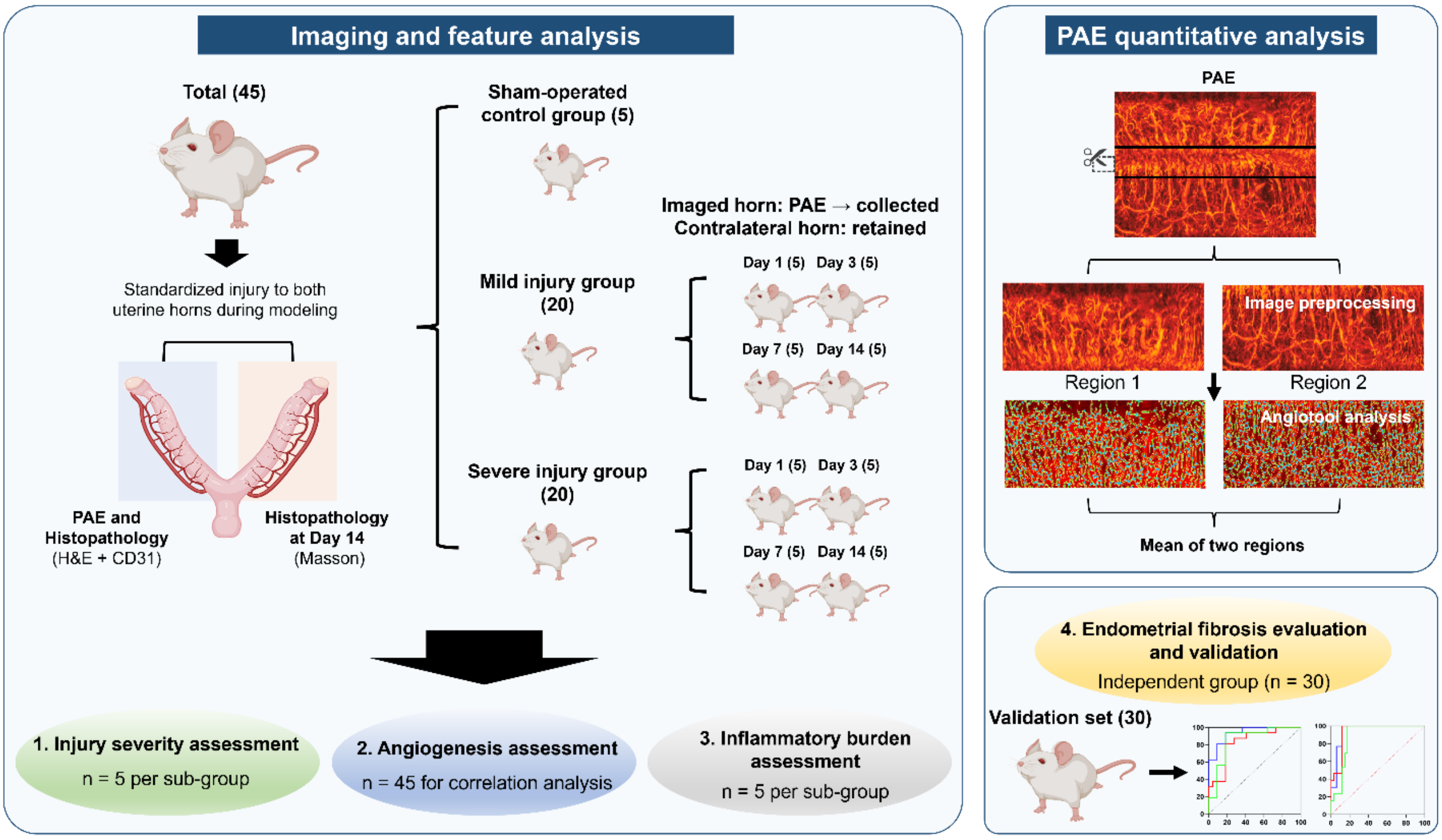

Figure S6. **Schematic of study design, PAE image processing and animal allocation.**

Table S1. Inter-observer reproducibility of quantitative PAE features.

PAE feature	ICC	95% CI
Vessel area	0.831	0.702-0.907
Vessel length	0.940	0.890-0.968
Number of junctions	0.918	0.851-0.956
Number of end points	0.939	0.887-0.967
Mean E lacunarity	0.870	0.768-0.929

ICC: intraclass correlation coefficient. CI: confidence interval
